# Impact of Transanal Drainage Tube Placement on Anastomosis Leakage Incidence After Rectal Cancer Surgery

**DOI:** 10.3390/life16010005

**Published:** 2025-12-19

**Authors:** Maria-Manuela Răvaș, Marian Marincaș, Eugen Brătucu, Vrgiliu Prunoiu, Laurentiu Simion, Laura-Maria Manea, Mircea-Nicolae Brătucu

**Affiliations:** 1 Clinical Department No 10, General Surgery, Carol Davila University of Medicine and Pharmacy, 050474 Bucharest, Romania; ravasmariamanuela92@gmail.com (M.-M.R.); mircea.bratucu@umfcd.ro (M.-N.B.); 2 Department of Oncological Surgery, Oncological Institute “Prof. Dr. Al. Trestioreanu”, 022328 Bucharest, Romania; 3 Department of Pneumopthiology, National Institute of Pneumophtiology “Marius Nasta”, 050159 Bucharest, Romania; 4 Department of General Surgery, Carol Davila Nephrology Clinical Hospital, 010731 Bucharest, Romania

**Keywords:** anastomotic leakage, septic complications, meta-analysis, retrospective studies, rectal cancer, transanal drainage tubes

## Abstract

**Background**: Anastomotic leakage (AL) following rectal cancer surgery is a significant cause of mortality and morbidity. Although transanal drainage tubes are expected to reduce the rate of AL, their preventive effect remains controversial. **Aim**: To evaluate whether transanal drainage tube (TAD) provides protection against AL in patients without other protective methods after low anterior resection (LAR). **Methods**: A retrospective cohort study was performed in patients undergoing LAR for rectal cancer between 2018 and 2023. Based on postoperative management, patients were divided into four distinct groups as follows: in TAD group, after colorectal anastomosis, a 32F silicone tube was inserted through the anus by more than 5 cm above the anastomosis. The tube was secured around the anus with a skin suture and a drainage bag was attached. The tube was removed after 3–5 days after surgery. In the non-TAD group, no transanal drainage tube and no diverting stomas, respectively, were used after the anastomosis. In the ileostomy and colostomy group a stoma was often performed as a primary measure in preventing anastomotic leakage. Clinical characteristics and postoperative complications were compared among the groups. Complications were categorized as general (eventration, seroma) or septic (fistula, abscess) and further classified as early (<7 days after surgery) or tardive (between 7 and 30 days after surgery). Statistical significance was defined as a *p*-value < 0.05. **Results**: A total of 171 patients were included: 47 (27.5%) in the TAD group, 54 (32.2%) in the non-TAD group, 25 (14.6%) in colostomy group, and 45 (26.3%) in ileostomy group. Overall, eight patients (4.7%) developed anastomotic leakage (AL). In the non-TAD group, 3 patients experienced AL (all early); in the ileostomy group, 2 patients (1 early, 1 tardive); and in the colostomy group, 2 patients (both tardive). The TAD group had one patient with AL as a tardive complication. The incidence of early general complications was significant lower in TAD group compared with the non-TAD group (OR 0.23, 95% CI [0.06–0.85]; *p* = 0.004), while there was no significant difference in early septic complications between TAD and ileostomy group (*p* = 0.71). The incidence of tardive general complications was significantly more frequent in the ileostomy group (OR 0.10, 95% CI [0.02–0.44]; *p* = 0.0008) compared with TAD group. Overall, total complications were significantly lower in TAD group compared to non-TAD (OR 0.15, 95% CI [0.05–0.44]; *p* < 0.001), ileostomy (OR 0.20, 95% CI [0.07–0.56]; *p* = 0.003), and colostomy ((OR 0.46 CI [0.21–0.99]; *p* = 0.049) groups. Furthermore, the TAD group showed a reduction rate of AL compared to the ileostomy, colostomy, and non-TAD groups (2.12% vs. 4.4%, 8%, and 5.5%) but the incidence of AL was almost similar (*p* = 0.65). Conclusions: The elective use of TAD is a simple and effective protective method for the prevention of overall postoperative complications, also helping to reduce the rate of AL in patients. Nevertheless, there is limited information in the literature regarding the optimal size and material of TAD, despite these factors playing an important role in the viability and effectiveness of the method.

## 1. Introduction

Colorectal cancer (CRC) is the third most commonly diagnosed cancer in the world, accounting for approximately 9.6% of all cancer cases, and remains the second leading cause of cancer-related mortality, according to the GLOBOCAN study [1]. The American Cancer Society’s estimates around 46.950 new rectal cases (RC) in 2025 (27.950 women and 19.000 men). Survival outcomes in RC are influenced by multiple factors including pathological staging, age, sex, along with the effectiveness of treatments like chemo-radiotherapy and surgery [2].

Low rectal cancer (LAR) constitutes a significant proportion of CRC cases. Older reports estimated ranges from 27% (in 1995) to 30–35% in more recent data, indicating either a shift in tumor distribution or improved screening protocols [3]. Surgery has been improved in management of rectal cancer, especially with minimally invasive approaches (laparoscopic, robotic); however, postoperative complications, especially anastomotic leakage (AL) remain a major clinical challenge. AL, with reported rates in the literature ranging from 1.3% to over 23%, can be associated with serious consequences such as peritonitis, sepsis, impaired functional recovery, prolonged hospital stay, and increased healthcare cost. The use of protective methods is frequently employed, particularly for distal anastomosis (<6 cm); thus, ileostomy is most common method [4].

Although several meta-analyses have reported that transanal drainage tubes (TADs) may reduce the incidence of AL after low anterior resection (LAR), important limitations remain in the existing evidence. Most published studies are heterogenous, thus pooling data from different surgical techniques, and often lack on reporting device characteristic (material and diameters), or standardized protocols placement and removal. Moreover, limited data about how to select the patients with highest risk for AL or whether specific TAD design confer a superior protective benefit.

Certain studies indicate that a diverting stoma can help to reduce life-threatening complications associated with AL, such as fecal peritonitis and sepsis; however, it does not lower the clinical incidence of leakage itself [5,6,7,8,9,10]. Overall, AL is a serious complication following colorectal surgery, associated with substantial mortality (7–12%) and morbidity (30%), and remains a key determinant of postoperative outcomes [11,12,13].

Therefore, the aim of this retrospective cohort study was to evaluate the efficacy of TAD as a protective method after LAR for rectal cancer. Only a few studies have compared TAD multiple protective methods including ileostomy, colostomy, and non-method—within the same cohort, and even fewer have examined the influence of tube design parameters (e.g., material, diameter) on clinical outcomes. To address these gaps, we analyzed a single-center cohort in which a TAD device (32F silicone tube) was consistently used, thereby reducing variability related to tube characteristics. The study included 171 who underwent the Dixon surgery for rectal cancer patients between January 2018 to March 2024. We assessed the effectiveness of TAD by evaluating the incidence of postoperative complications, with a specific focus on septic complications, particularly if it influences the rate of anastomotic leakage (AL). The summary of our findings is as follows.

## 2. Material and Methods

The study was designed as a retrospective, unicentric one, carried out on a cohort of 171 patients diagnosed with rectal cancer in the surgery department of the Bucharest Oncological Institute, “Prof. Dr. Alexandru Trestioreanu” (IOB), followed from 1 February 2018 the year in which the patients underwent surgery for a period of 5 years, until 1 February 2023. We included in the study all the patients who were diagnosed with rectal cancer, followed by low anterior resection with anastomosis between 5 and 15 cm above the anal verge.

### 2.1. Study Strategy

The transanal drainage tube (TAD) was introduced to our hospital on 1 February 2018. Patients who underwent rectal surgery with primary anastomosis at our institute were assigned to one of the following four groups: ileostomy, colostomy, TAD, and no-protection (non-TAD) groups.

In TAD group patients were managed with a transanal drainage tube following rectal cancer surgery, especially anastomosis located under the Douglas pouch.

The colostomy group included patients with circumferential cancer who had undergone a diverting colostomy prior to neoadjuvant therapy and the stoma was maintained for protective purposes.

The no-protection (non-TAD) group consisted of patients without any protective methods for the anastomosis.

The ileostomy group included patients who underwent diverting stoma when the anastomosis was located below the peritoneal reflection (under the Douglas pouch).

Patients who received neoadjuvant chemotherapy and/or radiation therapy were included only if they ultimately underwent anterior resection with primary anastomosis. Individuals with a complete remission were managed non-operatively with “watch-and-wait” approach. Patients with ultra-low tumors location (<4 cm from the anal verge), requiring abdominoperineal resection, were excluded, as no anastomosis was performed in these cases.

### 2.2. Inclusion and Exclusion Criteria

The inclusion criteria for this study were as follows: (1) tumors location between 5 cm and 15 cm from the anal verge; (2) patients undergoing anterior resection for rectal cancer, followed by anastomosis; (3) no diverting stoma was performed if the tumor was located above the Douglas pouch; (4) availability of complete clinical information including demographics, characteristics treatment details, and postoperative outcomes.

The exclusion criteria were as follows: (1) laparoscopic surgery during the study period, the number of laparoscopic anterior resections was too low to allow meaningful subgroup analysis, and these patients were excluded to ensure methodology consistency within a predominantly open surgery cohort; (2) palliative surgery with preoperative distant metastases; (3) patients who received neoadjuvant chemotherapy and/or radiation therapy and the tumor disappeared “wait and watch” strategy was applied; (4) patients where the tumor was in criteria for transanal local excisions; (5) patients with tumor locations less than 4 cm from anal verge that was mainly followed by abdominoperineal resection (APR); (6) surgery procedures that did not involve colorectal anastomosis, including Hartmann’s surgery, APR, and transanal partial resection.

### 2.3. Data Extraction

The extracted information included the type of surgery, type of anastomosis, type of drainage tube, tumor locations, number of patients who underwent chemo-radiotherapy, number of stomas, number of AL cases, number of patients non-TAD methods, and classification of AL, early and tardive septic complications, early and tardive general complications, age, gender, and mortality outcomes.

### 2.4. Treatment

The principles of surgery are respected. All surgeries were performed by at least 2 surgeons from same department. The surgeon for the TAD group is the same for all patients, while the surgeons in the other groups may differ. The neoplasm was staged by magnetic resonance imaging (MRI) of the pelvis and computer tomography (CT) examination of the chest and abdomen. The choice of APR or LAR was made after the analysis of preoperative imaging regarding the height of the tumor and the distance from the anal verge. The bowel is prepared prior to the surgery with antibiotics to prevent infection, as well as the administration of an osmotic laxative Fortrans. Total mesorectal excision (TME) is performed and it was mainly an open surgery. The circular anastomosis was inserted transrectally and the anastomosis was preformed endoluminally.

**Figure 1:**
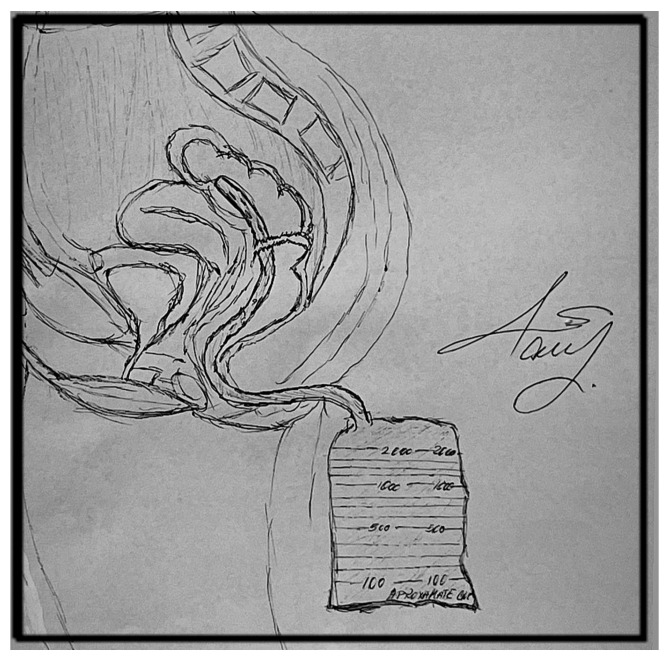
Illustration of the transanal tube (drawn by Maria M. Răvaș).

In the TAD group a 32F silicone tube was inserted through the anus, by more than 5 cm above the anastomosis. We initially used a 24F drainage tube with no benefits; therefore, a 32F tube was used for all patients. The tube was secured around the anus with silk 2-0 sutures, and a drainage system was attached (Figure 1). The TAD was kept in place for at least one week, with no reported intolerance of the tube. The non-TAD group had no protective methods following anastomosis. In the ileostomy and colostomy group, a stoma was performed right after the anastomosis was executed.

### 2.5. Outcomes

We are interested in the following outcomes listed below within 30 days postoperatively. The primary endpoint of this study was the occurrence of AL within 30 days after LAR in all groups. The secondary endpoint was to compare the incidence of overall postoperative complications within 30 days after surgery among the four groups (TAD, non-TAD, ileostomy, and colostomy). Complications were classified as early and tardive. Complications occurring in less than 7 days in patients who underwent LAR were defined as early and those occurring in more than 7 days were classified as tardive complications. The AL was defined according to current guidelines and diagnosed by clinical examination, laboratory markers, and radiologic imaging when indicated. Grade A anastomotic leakage requires no change in patient management or therapeutic intervention; grade B is clinically significant and requires active therapeutic intervention; and grade C requires a second abdominal surgery.

### 2.6. Statistical Analysis

The above statistical analysis was performed using the Statistical Package for the Social Sciences (SPSS) program, version 26.0 (IBM Corp., Armonk, NY, USA) software. To evaluate the distribution of continuous variables between groups, the Mann–Whitney U test was used, because they were not normally distributed. Categorical variables and nominal variables including postoperative complications, anastomotic leakage, mortality, and diversion type, were analyzed using Fisher’s exact test due to expected small cell count in several categories. The characteristics of the groups were compared after propensity score matching (PSM). In this study, the following variables were selected: sex, anastomosis distance from the anal verge, type of anastomosis, mortality rates, neoadjuvant chemotherapy, and TNM stages. Statistical significance was defined as *p* < 0.05.

## 3. Results

### 3.1. Patient Characteristics

A total of 171 patients were enrolled in the study 47 (27.5%) patients with trans anastomotic tube, 45 (26.3%) with ileostomy, 25 (14.6%) with colostomy, and 54 (32.2%) patients were in the non-protection group. The study population was predominantly male (65.49%). Most tumors were located within 10 cm from the anal verge (48.53% between 5 and 10 cm), and grade III (73.10%) was the most frequent tumor, followed by grade II (39.18%). The most common anastomosis technique was mechanical termino-terminal (TT). Anastomotic leakage (AL) occurred in 8 patients (4.67%); 1 patient in TAD group (2.12%)—tardive complications, 3 non-TAD group (all early complication 5.5%), 2 (4.4%) AL in ileostomy group (one early, one tardive) and it should be noted that one of this led to death of the patient; 2 colostomies, both tardive (8%). The overall incidence of fistula was 4.67% across all groups (TAD: 2.12%, non-TAD: 5.5%, ileostomy: 4.40%, colostomy: 8%).

Overall mortality rate was 1.75% (3/171), with deaths occurring in ileostomy, colostomy, and non-TAD groups. Notably, the death in the ileostomy group was due to anastomotic leakage. No deaths occurred in the TAD group. No deaths were recorded in the TAD group (Table 1).

### 3.2. Comparation of TAD vs. Non-TAD

Patients in the non-TAD group had more early general surgical complications compared to patients in the TAD group (OR 0.23, 95% CI [0.06–0.85], *p* = 0.004), suggesting a possible protective effect of the TAD methods. Total complications were also statistically higher in the non-TAD group reinforcing the importance of TAD (non-TAD= 33.3% vs. TAD= 10.6%, *p* = 0.0085). No statistically significant difference was observed for early septic complications (*p* = 0.121) or tardive complications, although there was a trend towards fewer tardive general complications in the TAD group (OR 0.27, 95% CI [0.05–1.32], *p* = 0.100). Total complications were significantly lower in TAD group compared to non-TAD (OR 0.15, 95% CI [0.05–0.44], *p* < 0.001) (Table 2).

### 3.3. Comparation of TAD vs. Ileostomy

Regarding early septic complications occurring within less than 7 days after the surgery, no statistical differences were found between the ileostomy and TAD group. However, the ileostomy group showed a significantly higher rate of tardive general complications (OR 0.10, 95% CI [0.02–0.44], *p* = 0.0008). Similarly, the odds of tardive septic complications were lower in the TAD group (*p* = 0.36), with no statistical difference. For early septic complications, the odds were lower in the TAD group (OR 0.18, 95% [0.01–3.32] *p* = 0.24) without statistical difference between the two groups. Total complications were significantly lower in the TAD group compared to ileostomy (OR 0.20, 95% CI [0.07–0.56], *p* = 0.003) (Table 3).

### 3.4. Comparation of TAD vs. Colostomy

The odds ratio [OR] of total complications was significantly higher in the colostomy group compared to the TAD group (OR 2.15, 95% CI [1.01–4.60], *p* = 0.0490), as a tardive complication associated with a stoma (OR 0.33 95% CI [0.04–2.91], *p* = 0.03). Total complications were significantly lower in the TAD group compared to colostomy (OR 0.46 CI [0.21–0.99], *p* = 0.049 (Table 4).

## 4. Summary

Overall, TAD placement was associated with significantly lower rates of total complications compared to non-TAD, ileostomy, and colostomy groups. TAD also demonstrated a protective effect against early general surgical complications (vs. non-TAD) and tardive general complications (vs. ileostomy). Although septic complications were generally less frequent in the TAD group, most comparations did not reach statistical significance, likely due to low event counts.

These results indicated that TAD is an independent protective factor, particularly for total and tardive general surgical complications.

## 5. Discussion

Anterior resection with total mesorectal excision is considered the gold standard for rectal cancer treatment. However, colorectal anastomosis located closer to the anal verge (<4 cm) is associated with a higher risk of anastomotic leakage (AL), one of the most serious complications in colorectal surgery. AL is defined as a defect in the intestinal wall at the anastomosis, including the sutures or staple line of neo-rectal reservoirs, which creates communication between the intra- and extraluminal compartments. Tumor location has been identified as an important independent risk factor for postoperative AL. Studies have shown that a tumor located less than 5 cm from anal verge is 6.5 times more likely to develop AL after surgery [14]. The severity of AL is classified according to the international study group of rectal cancer based on its impact on clinical management as follows: Grade A leakage does not require changes in patient treatment; Grade B leakage requires active therapeutic intervention but can be managed without re-laparotomy; and Grade C re-laparotomy is required [5].

Previous meta-analysis has shown contradicting results regarding the protective role of transanal drainage tube (TAD). A total of 1.259 patients found no significant difference in the incidence of Grade B AL or Grade C AL between the TAD and non-TAD groups; thus, TAD has not significantly affected the occurrence of AL (Grade B or C) following the surgery.

In the present study, we evaluated 171 patients undergoing low anterior rectal resection (LAR) for rectal cancer and examined the impact of TAD on postoperative complications incidence, with a particular focus on AL. The results showed that the incidence of AL was lower in the TAD group compared with the other three groups (non-TAD, ileostomy, and colostomy). Although the difference was not statistically significant, the results suggest a potential protective effect of TAD, particularly in reducing early septic and overall complications compared with non-TAD. In contrast, the patients without any protective method experienced higher early general complications with more total septical complications, supporting the possible protective effect of TAD in postoperative management.

Total complications were also statistically higher in the non-TAD reinforcing the importance of a TAD. Reflecting the effectiveness of non-operative management, drenage tube may provide protective benefits, but they do not entirely eliminate the risk of leakage, especially in high-risk patients. Additionally, TAD placement was not associated with increased complications such as anastomotic stenosis, bleeding, or infections [15].

According to the Dindo–Clavien classification, complications are categorized into several grades from I to V as follows: Grade I and II require pharmacological treatment; Grade IIIa is an intervention without general anesthesia and pharmacological treatment; Grade IIIb interventions with general anesthesia; Grade IVa and IVb life-threatening, multiple organ insufficiency; and Grade V death of the patient [16]. Based on the Dindo–Clavien classification, the overall complications in our study were Grade II, IIIa. Furthermore, there was a Grade V complication involving an early fistula (<7 days) despite ileostomy protection.

The incidence of fistulas, according to the literature, varies from 1.5% to 30%; up to one third of all postoperative mortality is due to AL, which also significantly increases morbidity and recurrence rate [17,18,19,20]. Overall fistula incidence was 4,6% with one Grade C leak (<7 days) that required re-laparotomy in the ileostomy group. However, the patients who performed ileostomy as a protective method demonstrated a significantly higher rate of tardive general complications compared with TAD. Meanwhile, transanal drainage tube was confirmed to be an independent protective factor for AL; it did not completely prevent postoperative fistula and one patient still experienced Grade B anastomotic leakage.

The mechanism contributing to AL is multifactorial. Technical challenges including tumor location (e.g., below the peritoneal reflection), mechanical errors, and patient-related comorbidities such as microvascular disease including hypertension, diabetes, obesity, smoking, radio-chemotherapy, ischemic heart disease are known as contributing factors for increasing the risk of complications. The absence of indocyanine-green fluorescence imaging, which can provide enhancement for better visualization of rectal wall vascularization, especially in high-risk patients, may have contributed to different outcomes. In our study, all patients who developed fistula had diabetes and hypertension, suggesting compromised microvascular status as a potential contributing factor.

Other significant risks factors include increased intraluminal pressure due to a reduced neo-rectal volume and decreased capacity. The rectum’s primary role is to store feces until an appropriate time of defecation; however, this function may be compromised or eliminated following rectal surgery. Proximal decompression can be effectively achieved through various clinical methods, including transanal tube or a diverting stoma [21].

Temporary loop ileostomy is the most common protective method for preventing the anastomotic leakage. Meta-analysis performed on eight studies that compared patients who had stoma versus no stoma indicates that following diverting stoma after LAR can reduce the AL without increasing mortality rates. Patients with stoma diversion experienced lower re-laparotomy rates compared to those without a stoma [22]. However, some researchers have emphasized that the anastomosis without a temporary diverting ileostomy does not impact long-term quality of life (QoL) [23].

Ileostomy itself can lead to numerous tardive complications, such as parastomal hernia, ileus, obstruction, wound infection, prolapse, retraction, stenosis, necrosis; in some cases, additional surgery under general anesthesia is required, thus exposing patients to further operative risks [24]. Our findings align with a previous study suggesting that ileostomy group has more tardive general complications with significant statical difference compared with the TAD group. Early septic complications were similar between the two groups; however, the ileostomy group experienced a Grade C leak (early complication) leading to death of the patient.

Notably, TAD was associated with a reduced rate of AL compared to the group without stoma protection (2.12% vs. 5.50%). Additionally, a meta-analysis involving 1.417 patients demonstrated that those without a diverting stoma can benefit from TAD as it reduces the rate of postoperative complications. Nevertheless, there was no significant difference in outcomes between the stomas and TAD groups [25].

Transanal drainage tubes can effectively prevent and reduce the incidence of AL, although there is limited information regarding the size tube and material [10,15,25,26,27,28].

TAD was developed with the aim of maximizing the decompressive effect. Its design includes a 32F silicone tube, with a diameter of 10.0 mm, wide openings, not easy to fold, shrink, or close, that facilitate effective drainage and decompression. Designed for easy insertion and removal, transanal tube features a curvilinear shape and a soft silicon, which help reduce the risk of injury to the rectum wall.

Limited studies compared different types of tubes with a diameter of 7.6 mm (24F) made of silicone and latex with the silicone tube with diameter of 10.00 mm (32F). Therefore, the 32F silicone tube demonstrated a significantly lower anastomotic leakage rate (AL) compared to the 24F latex and silicon tubes. Additionally, the time required for the first drainage and defecation after surgical intervention for rectal cancer was significantly shorter with the 32F silicone tube than with 24F silicon and latex tubes. These results showed that a 32F silicone tube has a superior drainage effect compared to the 24F silicone and latex tubes [29]. In our study, only the 32F silicone tube with a 10.0 mm diameter was used as the protective method. In few patients, we initially tested transanal tubes of a different diameter (24F) and materials; however, this alternative resulted in several inconveniences, including tube folding, lumen obstruction, and patient discomfort. Consequently, these tubes had to be removed before the recommended time. All patients from whom these tubes were removed were included in the non-TAD group.

Since TAD can have some advantages, it is an easy and fast procedure to perform; it can be removed without a surgery and it is safe and comfortable for patients. Despite these benefits, TAD cannot completely prevent AL, especially in high-risk patients. Nevertheless, compared with temporary stoma, TAD avoids stoma-related complications such as stenosis, prolapse, and necrosis; therefore, it remains a less invasive protective method.

Consequently, TAD could be a safe and simple alternative to diverting stoma in rectal surgery. Further large-scale or prospective randomized studies are necessary to provide more evidence for the broader clinical use of TAD.

The study presents several limitations. First, as a single-center retrospective study and relatively small sample size, issues such as confounders and some selection bias unavoidably existed: it used the propensity score matching (PMS) method to minimize between-groups bias.

Second, this study mainly included patients who underwent open surgery, and further exploration is needed in patients who undergo laparoscopic surgery.

Third, precise data on the exact height of the anastomosis from the anal verge were not available. After the excision of inferior rectal tumors, the decision regarding the appropriate distance for performing anastomosis or choosing rectal amputation vary among surgeons (for anastomosis <5 cm: 1–2 cm closer to the anal verge, as it increases the risk of complications). Since different surgeons performed the procedure for all methods, except in the TAD group, which was conducted exclusively by one surgeon, the variation in the experience and techniques among the surgeons could not be controllable within this study and may have influenced the outcomes.

Fourth, as we are not an emergency hospital, we lack control over patients who experienced severe complications and did not return to our hospital to report them; as a result, the follow-up relies on patients who revisit our clinic.

Finally, the depth and placement time of transanal tube may not be exactly the same for all patients, but it can be ensured to be >5 cm above the anastomosis, and kept at least 3 days; these variables need to be further explored.

Future prospective, multicenter, randomized trials, with standardized operative and follow-up protocols, are needed to confirm these findings (Table 5).

The study, in terms of sample size and exclusion of DS bias, explored the efficacy of TAD for prevention of postoperative AL.

## 6. Conclusions

A protective stoma has a non-negotiable complication rate. The consequences of anastomotic dehiscence after rectal surgery can put the patient’s life at risk; therefore, a protective method is needed especially when the anastomosis is located below the Douglas pouch.

The use of the transanal tube (TAD) requires a simple and feasible method for anastomosis protection, being a safe and effective alternative to conventional loop (ileostomy and colostomy, respectively). While diverting stoma possesses its own complications, TAD did not show any significant complications. Finally, we advocate for the use of a silicone tube with a 32F diameter and multiple side holes to ensure drainage.

Ultimately, further large prospective randomized studies are required to provide more evidence that can validate the expanded clinical use of TAD.

## Figures and Tables

**Table 1:** Clinicopathological characteristics.

Variable	Group A TAD (n = 47, 27.5%)	Group B ileostomy (n = 45, 23.6%)	Group C colostomy n = 25, 14.6%	Group D No protection n= 54, 32.2%	TOTAL 171
Gender, Male Female	26 (55.31%) 21 (47.72%)	35 (77.8%) 10 (22.2)	15 (60%) 10 (40%)	36 (66.7%) 18 (33.3%)	112 (65.59%) 59 (34.50%)
Radio-chemotherapy	38/35 (80.85%/74.46%)	43/38 (95.5%/84.44%)	21/20 (84%/80%)	30/31 (55.55%/57.40%)	135/124 (78.94%/72.51%)
Distance from anal verge (cm)					
I < 5 cm M5- < 10 cm S > 10 cm J	1 (2.1%)	10 (22.22%)	2 (8%)	8 (14.81%)	21 (12.28%)
	17(36.2%)	26 (57.8%)	19 (76%)	21 (12.3%)	83 (48.53%)
	23 (48.9%)	7 (15.6%)	3(12%)	16 (29.62%)	49 (28.65%)
	6 (12.76%)	2 (4.4%)	1 (4%)	10 (18.51%)	19 (11.11%)
Grade					
I	3 (6.4%)	6 (13.3%)	11 (44%)	6 (11.11%)	26 (15.20%)
II	18 (38.3%)	10 (22.2%)	23 (92%)	12 (22.22%)	54 (31.57%)
III	23 (48.9%)	24 (53.33%)	22 (88%)	27 (50%)	96 (56.14%)
IV	3 (6.38%)	2 (4.4%)	7 (28%)	9 (16.16%)	21 (12.28%)
TT/tt	18/10	27/1	15/0	14/2	(43.37%/7.60%)
LL/ll	1/0	7/0	3	2/1	(7.01%/2.30%)
LT/lt	3/11	10/0	7	6/1	(15.20%/11.11%)
Deads.	0	1 (2.20%)	1 (4.50%)	1 (1.85%)	3 (1.75%)
Anastomotic leakage (AL)	1 (2.12%)	2 (4.40%)	2 (8%)	3 (5.50%)	8 (4.67%)

LL—mechanical latero-lateral anastomosis. ll—manual latero-lateral anastomosis. TT—mechanical termino-terminal anastomosis. tt—manual termino-terminal anastomosis. LT—mechanical latero-terminal anastomosis. LT—manual latero-terminal anastomosis. Mortality: the deaths were recorded during the study. Ileostomy group: one death. Colostomy group: one death. Non-protection group: one death.

**Table 2:** Postoperative complications: TAD vs. non-TAD.

Group	TAD (n = 47)	NON-TAD (n = 54)	*p*-Value
Early General Surgical Complication	3 (6.40%)	11 (20.40%)	0.004
Early Septic Surgical Complications	0 (0%)	4 (7.40%)	0.121
Tardive General Surgical Complications	2 (4.30%)	8 (14.80%)	0.1003
Tardive Septic Surgical Complications	1 (2.10%)	1 (1.85%)	1
Total Septic Complications	1 (2.10%)	5 (9.25%)	0.0001
Total Complications	5 (10.60%)	18 (33.3%)	0.0085

**Table 3:** Postoperative complications: TAD vs. ileostomy.

Group	TAD (n = 47)	Ileostomy (n = 45)	*p*-Value
Early General Surgical Complication	3 (6.40%)	4 (8.9%)	0.711
Early Septic Surgical Complications	0 (0%)	2 (4.4%)	0.236
Tardive General Surgical Complications	2 (4.30%)	14 (31.10%)	0.00078
Tardive Septic Surgical Complications	1 (2.10%)	3 (6.7%)	0.356
Total Septic Complications	1 (2.10%)	5 (11.11%)	0.107
Total Complications	5 (10.60%)	17 (37.80%)	0.0031

**Table 4:** Postoperative complications: TAD and colostomy.

Group	TAD (n = 47)	Colostomy (n = 25)	*p*-Value
Early General Surgical Complication	3 (6.40%)	4 (16%)	0.207
Early Septic Surgical Complications	0 (0%)	1 (4%)	0.347
Tardive General Surgical Complications	2 (4.30%)	4 (16%)	0.173
Tardive Septical Surgical Complications	1 (2.10%)	1 (4%)	0.645
Total Septic Complications	1 (2.10%)	2 (4.44%)	0.275

**Table 5:** TAD and non-TAD studies.

Study [Ref]	Year	TAD Group	Controls Non-TAD	Type of Study	Anastomotic Leak %		*p* Value
					TAD	Non TAD	
Fujino et al. [25]	2023	489	486	Meta-analyze	4.5%	8.8%	0.012
Zhao et al. [26]	2013	81	77	Non-randomized	3.7%	10.3%	ns
Wang et al. [27]	2025	287	287	Retrospective	3.8%	8%	0.034
Xiao et al. [10]	2011	200	198	Randomized	2%	5%	0.026
Liu et al. [15]	2024	890	884	Meta-analyze	9.3%	10.3%	0.580
Nishigori et al. [28]	2014	36	140	Retrospective	9%	11.4%	0.040

## Data Availability

The patients’ data were obtained from the medical documents of the Bucharest Oncological Institute and they cannot be publicly available, as they contain personal and confidential data of the patients, but any information about these documents can be obtained on request from the corresponding authors.
